# The Lecture and the Lesson

**Published:** 1891-06

**Authors:** C. M. Wright

**Affiliations:** Cincinnati, O.


### The Lecture and the Lesson.

BY PROFESSOR C. M. WRIGHT, D.D.S., CINCINNATI, O.

Every reader of medical and dental periodical journals for a few years past, will have come to the conclusion that an opinion against the efficacy of the lecture system of teaching in our colleges has been steadily gaining ground. "The lecture system a failure" is like a party cry, and teachers, students, writers, and others, are gradually accepting the motto, without perhaps, serious thought upon the subject. The lecture system belongs to the past, it is claimed, and may have been well enough when the art of printing was in its infancy, before text-books became so common and so easily obtained. Again we hear that before books were published on a subject, the teacher's ideas and thoughts were only to be had in his lecture rooms, while now that knowledge is not an individual possession, and the mass of knowledge has been arranged in printed volumes, the student can acquire this by reading and by private study easier than by listening to a teacher, who may repeat only what may be found in the books. The teacher has no copyright on his lectures now, &c., &c. These are among the prominent reasons which seem to influence the general thought, and which are responsible for the gradual but steady change in the popular opinion. There may be other reasons, more particular and individual, which have helped the opposition; such as the kind of lectures inflicted upon the students, the dullness of lecturers, in style and thought, the inaptness of the student in the art of taking notes, the difficulty of following, by after study all the points given by the lecturer, the student not having time nor access to all the works on the subject, from which the professor has drawn his reason for the statements which he may make in his lecture. These are all legitimate reasons against the lecture system. Are they, however, sufficient to warrant the abandonment of a method that has for hundreds of years been in vogue, and that is so intimately associated with the knowledge and progress of to-day? Shall we cast away the ladder by which our father's climbed to the wisdom which they bequeathed to us, the same ladder by which we have advanced still higher, and to a point where we boast of our attainments? Shall we risk another and narrower, and more elementary method for our children, without very substantial reasons therefor? The opponents of the lecture have only the text book to offer in its stead, and in medical science we all know that text books can hardly keep pace with the facts and theories of the day. The advocates of the modern method lay great stress upon *recitation* on the part of the student. This practice is a necessity with the text-book lesson plan—and is often dispensed with where the lecture system prevails—but the lecture should not be confounded with the recitation, for in many colleges, medical and dental, the "quiz" is an established custom, and is conducted regularly either by the professor or by an appointed "quiz-master." Neither party denies the value of recitation on the part of the student, therefore there is no danger of this practice becoming obsolete.

Personal experiences in *methods* vary according to character and disposition of experimenters, even when all agree as to the final object of the methods—but I should like to offer the results of my own experience in teaching one branch of medical science, viz., physiology: For years the lecture and "quiz" had been the accepted method of teaching in this department of the Ohio College of Dental Surgery. Last year, owing to the reasons which I have stated in the beginning of this paper, I abandoned the lecture altogether and adopted the text-book system, in both junior and senior classes. Regular lessons were assigned and students were called upon by name to answer questions on the lessons of the day. The lecture room was turned into a school room, the young men were turned into school boys. They carried their books to and from school like boys; they studied so many pages for each lesson of the particular text-book adopted, and if a question was proposed by the teacher which referred to a subject intimately related to the one studied for that day, but not included between page — and page —, (the lesson for that day) the student immediately answered "'Tisn't in the lesson;" "We haven't got to that yet," &c. The teacher felt somewhat hampered by the lesson of the day. He was handicapped by the text-book, for to the student's mind everything was unimportant, that was not where he could put his finger on it in his book. The whole tendency of the method was to bind both teacher and pupil fast to one text-book. Where is the single text-book on any subject that the intelligent teacher is willing to swallow whole, or stick to, without even the privilege that the preacher has of expressing his individual views about the text of holy writ?

This narrowing and confining of the subject within certain limits, this restraint upon the teacher, who has felt at liberty to browse upon all text-books, all periodical literature, all sciences, for his points and illustrations, is an objection which presents itself with force to the mind of the teacher himself.

This, I am well aware, would not be an argument in favor of the lecture which could not be answered by a single question, viz.: Is it for the pleasure or benefit of the teacher, or for the benefit of the pupil that methods of instruction are adopted? Now let us turn our attention to the student. Does he get as much from the pages of one text-book as he would from a lecture, illustrated, simplified, made forcible by repetitions, by anecdote, and impressed upon him by the living words of a live teacher? Does the student not lose something of value when he has not the opportunity of acquiring habits of attention to spoken words and habits of note-taking. Is it a fact that clear perceptions are more easily or more surely made by impressions of printed words upon the retina than by impressions of vocal sounds upon the end organs of the special sense of hearing? As a matter of fact, can we not rather assert that both stimulations of the perception are valuable, and that one cannot be discarded without the loss of power, especially where numbers of minds are concerned?

Personally, from past experience in the lecture room with classes of dental students, and from last year's experiment with the text-book lesson plan, I feel convinced that neither method exclusively followed, will attain the best results. That a combination of the *lecture* and the *lesson* will more nearly meet the requirements.

I should then advise, as worthy of trial, the following plan (subject to variations to meet individual views): First, the adoption of a text-book, and the assigning of regular lessons. These lessons to be learned by the student, who must be prepared to recite them when called upon.

Second: A lecture upon the same subject at another regular meeting, reviewing, condensing, illustrative, explaining, etc., in character, during which the student is expected to take notes, which will form a part of his text book, or which he may regard as a work of reference.

Thirdly: Reviewing recitations, when the student can reproduce from memory the images he has taken so much pains to impress upon the gray cells of this part of his cerebrum—by the study of his text-book and his note-book.

There are psychological and physiological reasons for this method—this combination of methods—which I shall not now take the time to discuss, but which will appeal to all, when we remember the structure and functions of the special sense organs and their relation to the brain, and when we consider the modus operandi of the mind in relation to knowledge.

---

"Dental journalism, therefore, found the springs of its birth in a desire to supplement the early meagre college training and has ever since drawn its life from the veins of a profession to which it relates as a post-graduate course."

DR. L. ASHLEY FAUGHT.

